# Oxidative-Inflammatory Stress in Immune Cells from Adult Mice with Premature Aging

**DOI:** 10.3390/ijms20030769

**Published:** 2019-02-12

**Authors:** Antonio Garrido, Julia Cruces, Noemí Ceprián, Elena Vara, Mónica de la Fuente

**Affiliations:** 1 Department of Genetics, Physiology and Microbiology (Animal Physiology Unit), School of Biology, Complutense University of Madrid (UCM), 28040 Madrid, Spain; j.cruces@pdi.ucm.es (J.C.); nceprián@gmail.com (N.C.); mondelaf@bio.ucm.es (M.d.l.F.); 2 Institute of Investigation of Hospital 12 de Octubre (i+12), 28041 Madrid, Spain; 3 Department of Biochemistry and Molecular Biology III, School of Medicine, Complutense University of Madrid (UCM), 28040 Madrid, Spain; evaraami@med.ucm.es

**Keywords:** premature aging, oxi-inflamm-aging, immunosenescence, leukocytes, PAM, TH-HZ mice

## Abstract

Oxidative and inflammatory stresses are closely related processes, which contribute to age-associated impairments that affect the regulatory systems such as the immune system and its immunosenescence. Therefore, the aim of this work was to confirm whether an oxidative/inflammatory stress occurs in immune cells from adult mice with premature aging, similar to that shown in leukocytes from chronologically old animals, and if this results in immunosenescence. Several oxidants/antioxidants and inflammatory/anti-inflammatory cytokines were analyzed in peritoneal leukocytes from adult female CD1 mice in two models of premature aging—(a) prematurely aging mice (PAM) and (b) mice with the deletion of a single allele (hemi-zygotic: HZ) of the tyrosine hydroxylase (*th*) gene (TH-HZ), together with cells from chronologically old animals. Several immune function parameters were also studied in peritoneal phagocytes and lymphocytes. The same oxidants and antioxidants were also analyzed in spleen and thymus leukocytes. The results showed that the immune cells of PAM and TH-HZ mice presented lower values of antioxidant defenses and higher values of oxidants/pro-inflammatory cytokines than cells from corresponding controls, and similar to those in cells from old animals. Moreover, premature immunosenescence in peritoneal leukocytes from both PAM and TH-HZ mice was also observed. In conclusion, adult PAM and TH-HZ mice showed oxidative stress in their immune cells, which would explain their immunosenescence.

## 1. Introduction

Aging is characterized by a general and progressive deterioration of the physiological functions of the organism with a lessened capacity to maintain homeostasis and, consequently, an increase in morbidity and mortality. Thus, although aging is not a disease, the age-related function impairment, increases the risk of the loss of health. Indeed, the deterioration of the homeostatic systems: the nervous, endocrine and immune systems as well as of the cross-talk between them, namely neuroimmunoendocrine communication, which allows the maintenance of health, are especially involved in the risk of morbidity with aging [1]. In fact, the immune system, with advanced age, suffers numerous changes that affect the innate and acquired immune responses, which are called immunosenescence [2,3,4], and these have been related to the risk of suffering infections, autoimmune diseases and cancers [3,5,6,7]. In this context, several immune system parameters have been related to health and risk of mortality [5,8,9]. Moreover, several functional capacities of immune cells have been proposed as markers of the rate of aging of an individual and as predictors of life expectancy [10]. 

Among the more than 400 theories published that try to explain the aging process, one of the most widely accepted is the theory of free-radicals, proposed by Harman [11] and further developed by several researchers [12,13,14,15]. This theory indicates that aging is a consequence of the accumulation of damage by deleterious oxidation in biomolecules caused by the high reactivity of the free radicals produced in cells as a result of the necessary use of oxygen. Although several authors reject this idea [16,17], the redox theory of aging appears to be accepted by many others [12,13,14,15]. Another relevant theory is that of “inflamm-aging” [18,19,20], which points out the presence of a mild inflammation in aging. More recently, the oxidation-inflammation theory of aging was proposed to combine the age-related increases of oxidation and inflammation [12], two intimately related processes [21,22]. This theory suggests that the age-related changes in the organism are closely linked to chronic oxidative-inflammatory stress, which damages the cellular components, including proteins, lipids and DNA. This affects all cells of the organism, but especially those of the homeostatic systems, as well as the communication between them, resulting in an impairment of their functions, and the consequent loss of homeostasis and health. This theory, in which the idea of “oxi-inflamm-aging” was suggested, also proposes a key involvement of the immune system in the rate of aging of the organism. The immune cells, due to their capacity to produce oxidant and inflammatory compounds in order to carry out their different functions, could, as a result of age-related oxidative damage, lose the capacity to regulate their own redox and inflammatory balances and consequently increase the oxidative-inflammatory stress of the organism. Moreover, this stress seems to constitute the basis of immunosenescence [12,23,24]. Nevertheless, the appearance of age-related changes in the immune system can be modulated. In fact, several potential interventions, such as hormonal replacement therapies, physical activity, cytokine therapy, and/or altering microbiota profiles, have been reported as capable of attenuating immunosenescence and preserving health [25,26,27]. However, other situations, such as an inadequate stress response, can contribute to the age-related changes that the immune system suffers, thus increasing the probability of early death. In fact, an altered stress response has been associated with premature or accelerated aging [28,29,30].

In addition, heterogeneity is an important characteristic of the aging process. Thus, the physiological state of each individual can be different in comparison with others of the same chronological age, thus giving rise to the concept of “biological age,” which shows the rate of aging of a subject [31]. Given that the biological age is a better indicator than the chronological age of the state of health, vitality and remaining active life expectancy of each individual [31,32], its determination is very relevant. However, although many studies have been carried out trying to obtain the most appropriate parameters for determining biological age, none of the sets of markers proposed have yet been completely validated. To validate these markers, experimental animals such as mice, with longevity shorter than that of humans, are needed. In fact, several years ago we proposed a model of prematurely aging mice (PAM), based on altered stress-related behavioral response, in which animals, at the adult age, showed a clear premature immunosenescence as well as signs of an oxidative stress establishment. These resulted in a shorter mean lifespan than that of the corresponding non-prematurely aging mice (NPAM) of the same sex and age [10,33,34]. In addition, female mice with the deletion of a single allele (hemi-zygotic: HZ) of the tyrosine hydroxylase (*th*) gene (TH-HZ), and with the consequent lower catecholamine content, even in their immune cells, presented a premature immunosenescence in their peritoneal leukocytes, exhibiting higher oxidative stress in these cells and having a lower mean lifespan in comparison to wild type (WT) mice [35]. However, whether these PAM and TH-HZ mice, at adult age, would show oxi-inflamm-aging in these leukocytes and those from other immunological organs, such as spleen and thymus, and whether they exhibited in their immune cells a pattern of functions and redox state with similar values to those observed in chronologically old mice, are aspects that have not been studied yet.

Therefore, the aim of the present work was to confirm the establishment of oxi-inflamm-aging in peritoneal, spleen and thymus leukocytes from both adult PAM and TH-HZ mice, which have been proposed as models of premature aging, and to determine whether their oxidative-inflammatory stress is similar to that observed in chronologically old mice. For that, we analyzed several anti-oxidant and anti-inflammatory defenses, as well as oxidant and pro-inflammatory compounds in peritoneal, spleen, and thymus leukocytes from PAM and TH-HZ mice, and their corresponding controls. Moreover, the values of all these parameters were compared with those obtained from chronologically old mice. In order to corroborate the establishment of immune senescence in these animals, several functions were studied in their peritoneal leukocytes. To determine the possible alteration in catecholamine homeostasis, in the case of PAM, endogenous peritoneal CA levels were also analyzed.

## 2. Results

**Figure 1:**
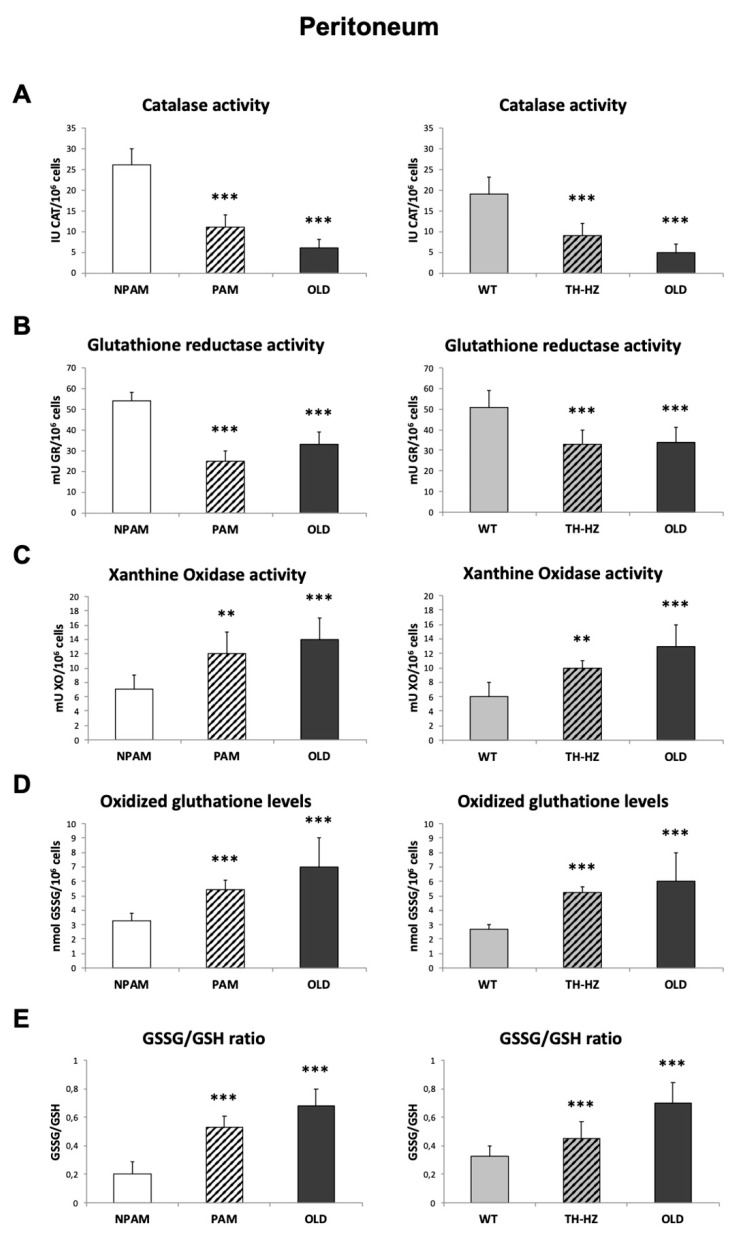
Parameters of redox state in peritoneal leukocytes. Anti-oxidant defenses: activity of catalase (CAT) (IU CAT/10^6^ cells) (**A**) and glutathione reductase (GR) (mU GR/10^6^ cells) (**B**); oxidants: activities of xanthine oxidase (XO) (mU XO/10^6^ cells) (**C**), concentrations of oxidized glutathione (GSSG) (mmol GSSG/10^6^ cells) (**D**), and redox balance (GSSG/GSH ratios) (**E**) in peritoneal immune cells from adult PAM, NPAM, TH-HZ, WT mice, and chronologically old animals. PAM: prematurely aging mice, NPAM: non-prematurely aging mice, TH-HZ: tyrosine hydroxylase hemi-zygotic mice, WT: wild-type mice. Each column is the mean ± SD of the values of 10 experiments corresponding to 10 animals. ** *p* < 0.01, *** *p* < 0.001 with respect to the corresponding controls.

A decrease in antioxidant defenses together with an increase in several oxidants have been observed to appear in peritoneal cells with age [21,36,37]. In the present study, the antioxidants and oxidants analyzed in these cells are shown in Figure 1. CAT and GR activities (Figure 1A,1B, respectively) were lower (*p* < 0.001) in cells from adult PAM and TH-HZ mice (36 ± 4 weeks of age) than in their corresponding controls (NPAM and WT, respectively), and similar to those in cells from old mice (84 ± 4 weeks), which also showed lower (*p* < 0.001) values than those in adult controls (NPAM and WT). The activity of XO, an enzyme considered oxidant, (Figure 1C) showed higher (*p* < 0.001) values in cells from old mice than in those of adult controls (NPAM and WT mice) and similar to those of PAM and TH-HZ mice. Furthermore, GSSG amounts were also higher (*p* < 0.001) in peritoneal cells from old mice and adult PAM and TH-HZ mice than in those of the corresponding NPAM and WT animals (Figure 1D). The same differences were obtained for the GSSG/GSH ratios (Figure 1E).

**Figure 2:**
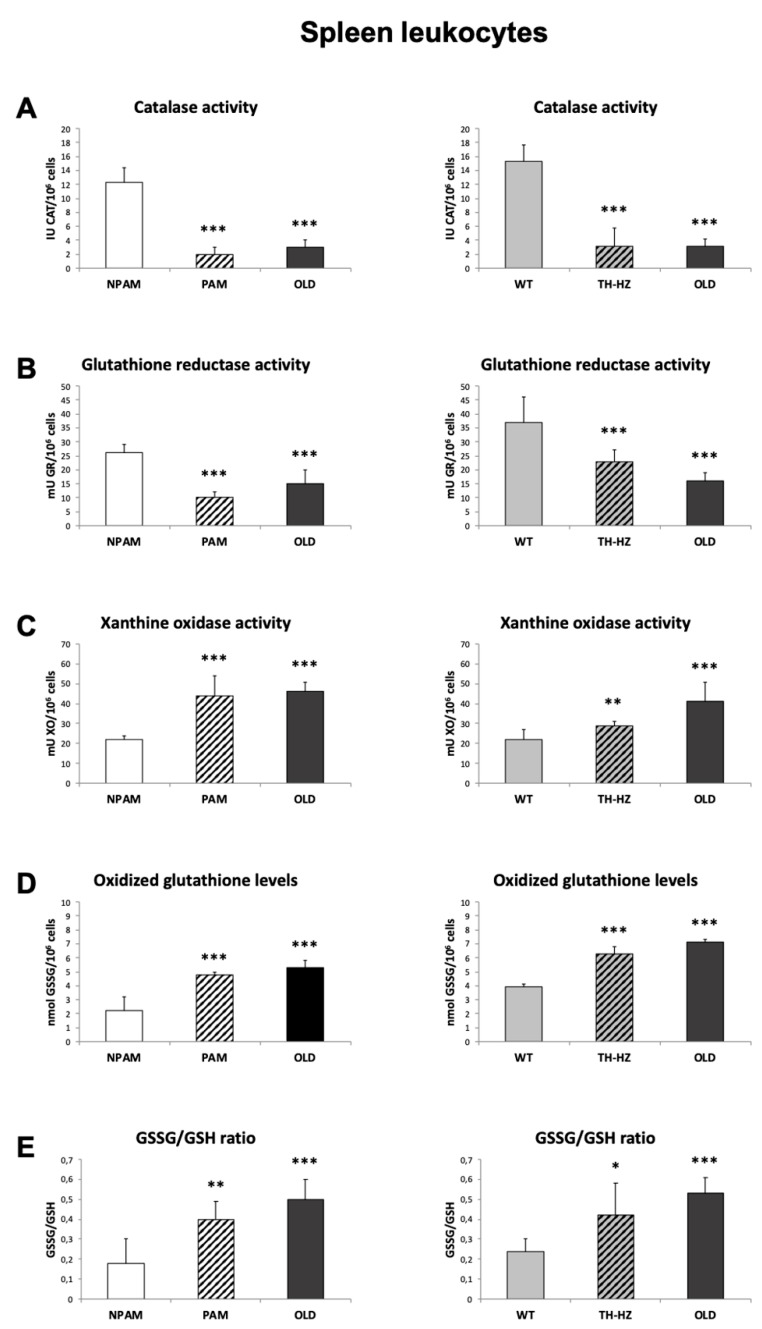
Parameters of redox state in spleen leukocytes. Anti-oxidant defenses: activities of catalase (CAT) (IU CAT/10^6^ cells) (**A**) and glutathione reductase (GR) (mU GR/10^6^ cells) (**B**); oxidants: activity of xanthine oxidase (XO) (mU XO/10^6^ cells) (**C**), and concentrations of oxidized glutathione (GSSG) (mmol GSSG/10^6^ cells) (**D**), as well as redox balance (GSSG/GSH ratios) (**E**) in spleen leukocytes from adult PAM, NPAM, TH-HZ, and WT mice and from old mice. PAM: prematurely aging mice, NPAM: non-prematurely aging mice, TH-HZ: tyrosine hydroxylase hemi-zygotic mice, WT: wild-type mice. Each column is the mean ± SD of the values of 10 experiments corresponding to 10 animals. * *p* < 0.05, ** *p* < 0.01, *** *p* < 0.001 with respect to the corresponding controls.

**Figure 3:**
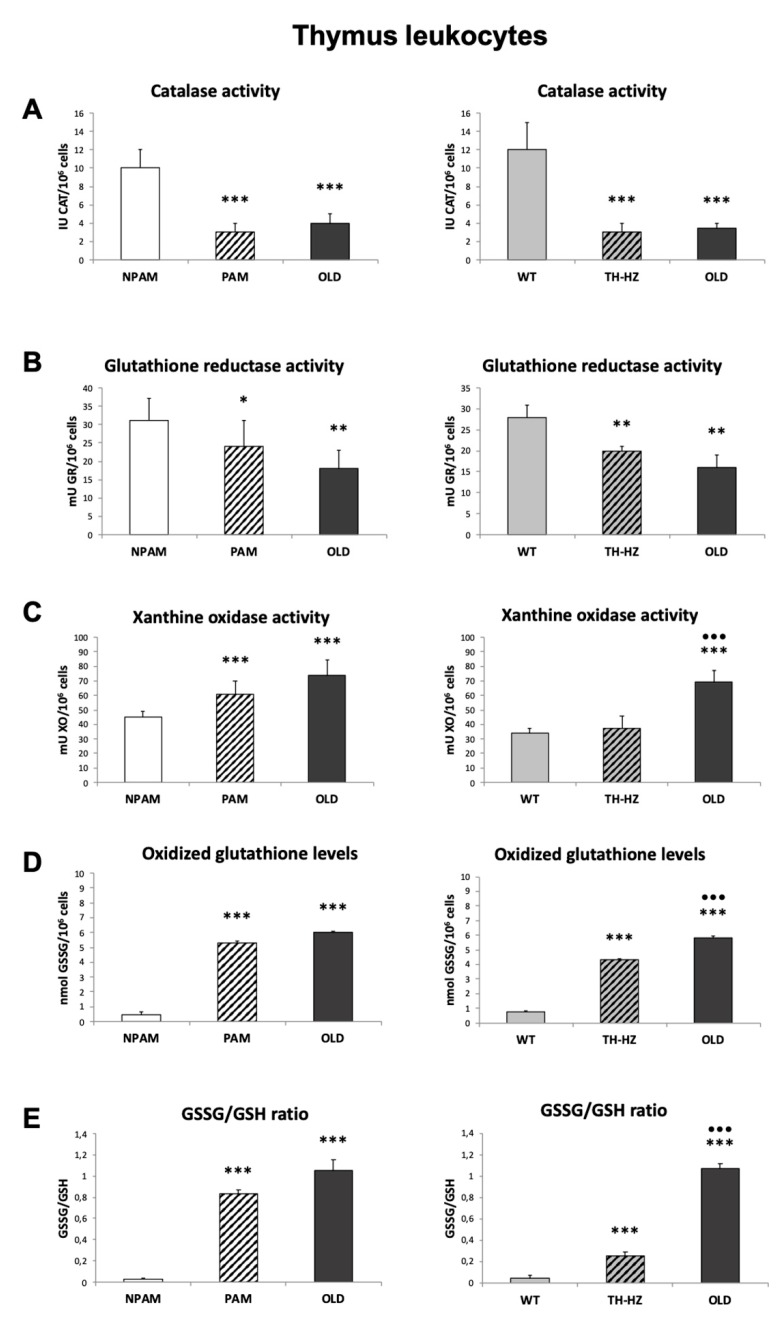
Parameters of redox state in thymus leukocytes. Anti-oxidant defenses: activity of catalase (CAT) (IU CAT/10^6^ cells) (**A**) and glutathione reductase (GR) (mU GR/10^6^ cells) (**B**); oxidants compounds: activity of xanthine oxidase (XO) (mU XO/10^6^ cells) (**C**) and concentrations of oxidized glutathione (GSSG) (mmol GSSG/10^6^ cells) (**D**) as well as redox balance (GSSG/GSH ratios) (**E**) in spleen leukocytes from adult PAM, NPAM, TH-HZ, and WT mice and from old mice. PAM: prematurely aging mice, NPAM: non-prematurely aging mice, TH-HZ: tyrosine hydroxylase hemi-zygotic mice, WT wild-type mice. Each column is the mean ± SD of the values of 10 experiments corresponding to 10 animals. * *p* < 0.05, ** *p* < 0.01, *** *p* < 0.001 with respect to the corresponding controls. ··· *p* < 0.001 with respect to PAM or TH-HZ.

With the objective to determine whether the oxidative stress observed in peritoneal leukocytes could be shown in other immunological locations, we analyzed several oxidants and anti-oxidant defenses in spleen and thymus from PAM, TH-HZ, their corresponding controls (NPAM and WT) and chronologically old mice. Both spleen and thymus leukocytes from PAM and TH-HZ mice presented lower values of the antioxidants studied (CAT and GR activities) and higher of the oxidants (XO activity, GSSG amounts and GSSG/GSH ratios) than those of NPAM and WT mice, as well as similar values to those in cells from old mice, with the exception of the XO activity in the thymus leukocytes from the TH-HZ mice (Figure 2 and Figure 3).

**Figure 4:**
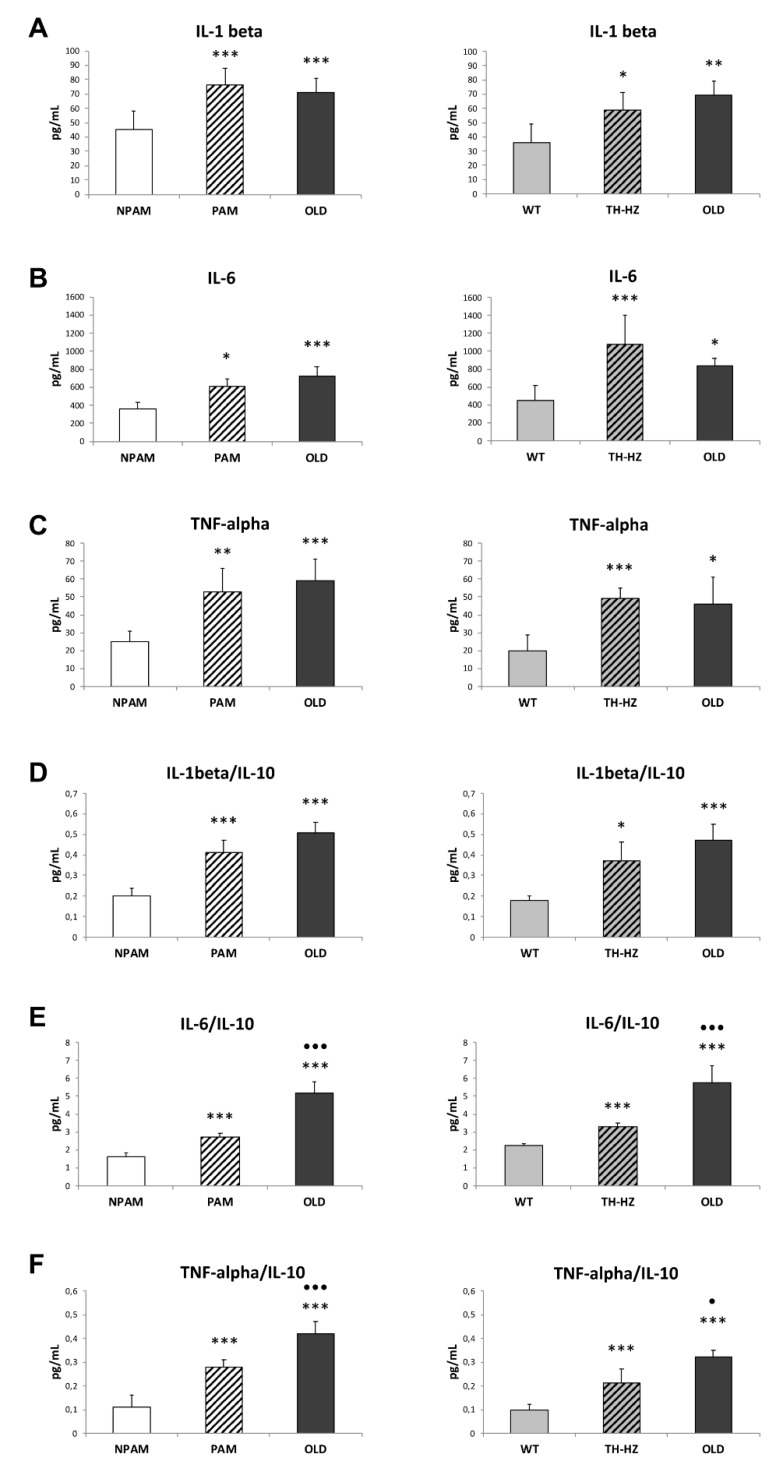
Pro-inflammatory cytokines (pg/mL) released by peritoneal leukocytes incubated in basal conditions, as well as pro-inflammatory/anti-inflammatory ratios. IL-1β (**A**), IL-6 (**B**), TNF-α (**C**), as well as IL-1β/IL-10 (**D**), IL-6/IL-10 (**E**), and TNF-α/IL-10 (**F**) ratios in adult PAM, NPAM, TH-HZ, WT mice, and in chronologically old animals. PAM: prematurely aging mice, NPAM: non-prematurely aging mice, TH-HZ: tyrosine hydroxylase hemi-zygotic mice, WT wild-type mice. Each column is the mean ± SD of the values of 10 experiments corresponding to 10 animals. * *p* < 0.05, ** *p* < 0.01, *** *p* < 0.001 with respect to the corresponding controls. · *p* < 0.05, ··· *p* < 0.001 with respect to PAM or TH-HZ.

Regarding to inflammatory stress, which shows an age-related increase [18,19,20,37,38,39,40], several pro-inflammatory cytokines such as IL-1β, IL-6, and TNF-α, as well as anti-inflammatory IL-10 released by peritoneal leukocytes incubated in basal conditions (without any stimulus), were analyzed. The concentrations of the pro-inflammatory cytokines released by peritoneal leukocytes from adult PAM and HT-HZ mice without stimulus were higher than those in adult controls (NPAM and WT) and similar to the concentrations found in cultures of cells from old mice (Figure 4). Nevertheless, not differences were observed between both experimental groups (PAM and TH-HZ mice; 189 ± 88 pg/mL and 161 ± 108 pg/mL, respectively) and their corresponding controls (NPAM and WT animals, 227 ± 23 pg/mL and 201 ± 50 pg/mL respectively) in the case of IL-10 levels, although the ratios of the pro-inflammatory/anti-inflammatory cytokines (Figure 4) were higher in PAM and TH-HZ mice (with the exception of IL-1β/IL-10 in TH-HZ) than in their respective NPAM and WT controls (Figure 4). Old mice presented the highest ratios in all cases.

**Figure 5:**
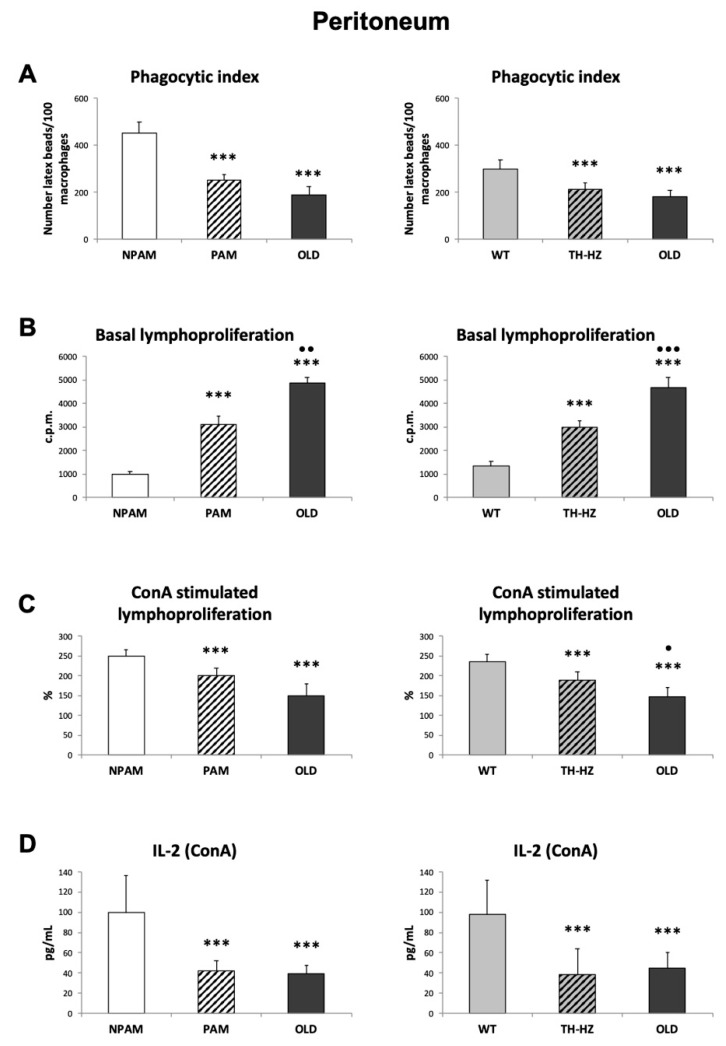
Immune functions in peritoneal leukocytes. Phagocytosis of macrophages (phagocytic index: number of latex beads ingested by 100 macrophages) (**A**); Basal lymphoproliferation (c.p.m.) (**B**); Stimulated lymphoproliferation in response to ConA (%) (**C**); IL-2 (pg/mL) released in presence of ConA (**D**) in peritoneal immune cells from adult PAM, NPAM, TH-HZ, WT mice and chronologically old animals. PAM: prematurely aging mice, NPAM: non-prematurely aging mice, TH-HZ: tyrosine hydroxylase hemi-zygotic mice, WT wild-type mice. Each column is the mean ± SD of the values of 10 experiments corresponding to 10 animals. ** *p* < 0.01, *** *p* < 0.001 with respect to the corresponding controls. · *p* < 0.05, ·· *p* < 0.01, ··· *p* < 0.001 with respect to PAM or TH-HZ.

The age-related oxidative stress in immune cells has been proposed as a cause of immunosenescence [12,23,41,42]. We previously observed that several functions of peritoneal immune cells, which decrease with aging [10,37], showed values in adult PAM and in TH-HZ lower than in NPAM and WT mice, respectively [29,30,31,43,44,45]. Because of this, we analyzed some of these functions such as the phagocytosis of macrophages, the proliferation of lymphocytes (basal and in response to ConA) and the IL-2 release in cultures with ConA, in peritoneal leukocytes of adult PAM and TH-HZ mice, and of the corresponding controls (NPAM and WT) as well as of those from old animals (a comparison not previously performed). The results are shown in Figure 5, and, in general, the values obtained in leukocytes from adult PAM and TH-HZ mice were similar to those of chronologically old animals, and lower than those in the corresponding adult controls.

The phagocytic capacity, the most typical function of macrophages, which decreases with age [12,40,46], was measured by the phagocytic index (number of latex beads ingested by 100 macrophages) (Figure 5A). The results showed that these numbers were lower (*p* < 0.001) in PAM and TH-HZ than in NPAM and WT, respectively, and were similar to those in cells from old animals. In the case of the results of basal lymphoproliferation, immune function that is increased with age due to chronic low-inflammation appearance [18,19,20,30,31,32,33,34,35,36,37,38,39,40,41], the c.p.m. in leukocytes from PAM and TH-HZ were higher (*p* < 0.001) than those in the corresponding controls (NPAM and WT, respectively). In leukocytes from old animals the c.p.m. were higher (*p* < 0.001) than those from the NPAM and WT, but also from those in PAM (*p* < 0.01) and TH-HZ mice (*p* < 0.001) (Figure 5B). However, the results of the percentages of stimulation of lymphoproliferation in response to mitogens such as ConA (Figure 5C), a capacity that clearly decreases with age [2,10,37,39,40,47], showed lower values (*p* < 0.001) in cells of old animals than in those of adult controls (NPAM and WT). The values obtained in adult PAM and TH-HZ were also lower (*p* < 0.01 and *p* < 0.001, respectively) than those in their corresponding controls and similar to those in old animals in the case of PAM, although higher (*p* < 0.05) in the case of TH-HZ mice. Furthermore, the release of IL-2 in presence of the mitogen ConA, which is closely related to the lymphoproliferative capacity, also decreases with age [2,10,37,39,40,47]. In fact, the results in the present study showed (Figure 5D) that leukocytes from old animals release lower (*p* < 0.001) amounts of IL-2 than those from adult controls (NPAM and WT), but similar to those released by PAM and TH-HZ, which were lower (*p* < 0.001) than those in their corresponding controls.

**Figure 6:**
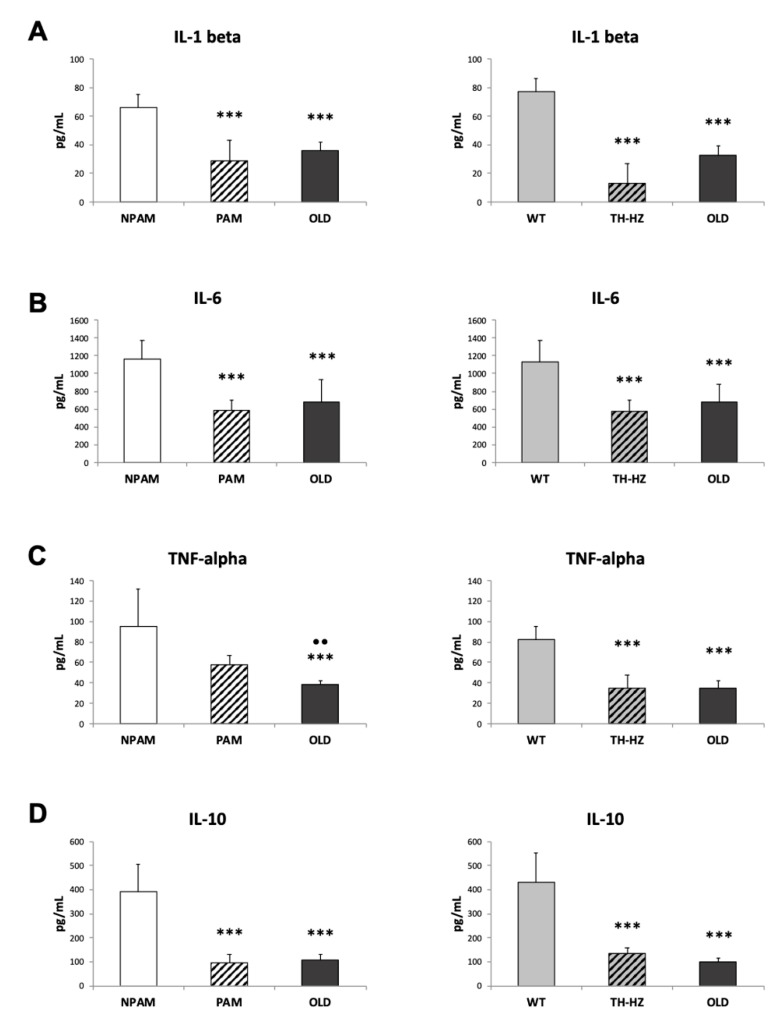
Pro-inflammatory and anti-inflammatory cytokines (pg/ml) released by peritoneal leukocytes incubated in presence of the mitogen ConA. IL-1β (**A**); IL-6 (**B**); TNF-α (**C**); IL-10 (**D**) released by cells from adult PAM, NPAM, TH-HZ, WT mice, and chronologically old animals. PAM: prematurely aging mice, NPAM: non-prematurely aging mice, TH-HZ: tyrosine hydroxylase hemi-zygotic mice, WT wild-type mice. Each column is the mean ± SD of the values of 10 experiments corresponding to 10 animals. * *p* < 0.05, *** *p* < 0.001 with respect to the corresponding controls. ·· *p* < 0.01 with respect to PAM.

**Figure 7:**
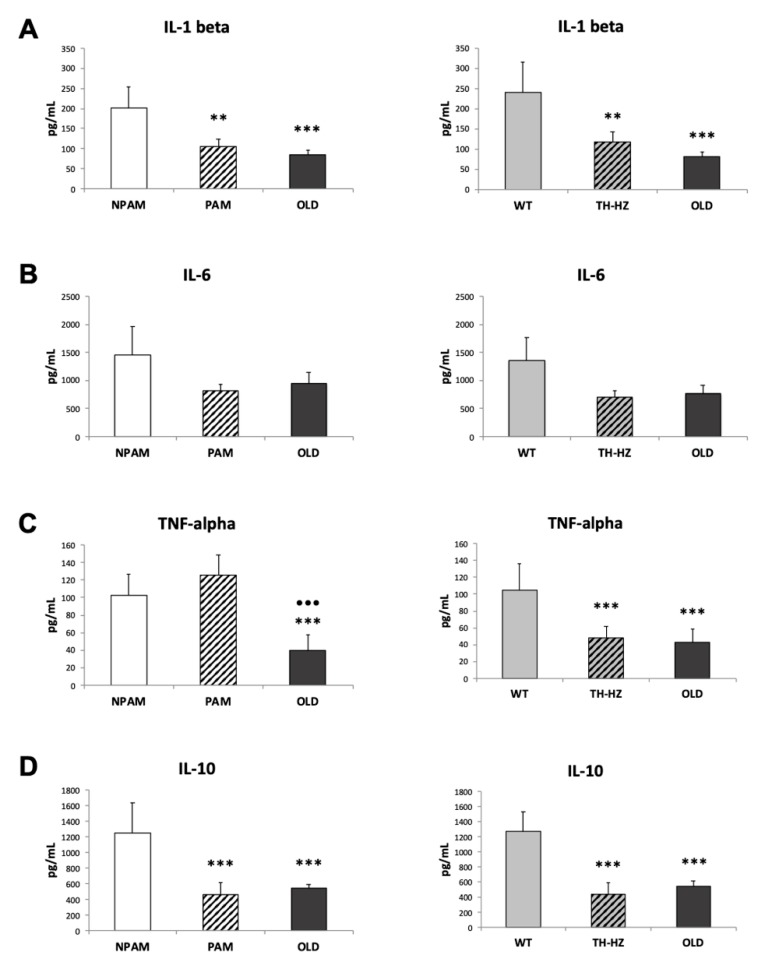
Pro-inflammatory and anti-inflammatory cytokines (pg/mL) released by peritoneal leukocytes incubated in presence of the mitogen LPS. IL-1β (**A**); IL-6 (**B**); TNF-α (**C**); IL-10 (**D**) released by cells from adult PAM, NPAM, TH-HZ, WT mice, and chronologically old animals. PAM: prematurely aging mice, NPAM: non-prematurely aging mice, TH-HZ: tyrosine hydroxylase hemi-zygotic mice, WT wild-type mice. Each column is the mean ± SD of the values of 10 experiments corresponding to 10 animals. ** *p* < 0.01, *** *p* < 0.001 with respect to the corresponding controls. ··· *p* < 0.001 with respect to PAM.

With the objective to analyze whether an inadequate immune response presented by cells from PAM and TH-HZ mice could be due to an altered cytokine network, we evaluated several pro-inflammatory (IL-1β, IL-6 and TNF-α) and anti-inflammatory IL-10 cytokine released by peritoneal leukocytes incubated in response to ConA and LPS. The results are shown in Figure 6 and Figure 7. All these cytokines were lower in PAM and TH-HZ peritoneal leukocytes incubated with ConA (Figure 6) than in those of their corresponding controls (NPAM and WT) with the exception of TNF-α in the PAM group. The old mice also secreted lower concentrations of cytokines in response to ConA than adult controls and, in general, similar to those in cells from PAM and TH-HZ mice. The concentrations of cytokines released in response to LPS (Figure 7) were generally lower in cells of PAM, TH-HZ and old mice with respect to those in NPAM and WT mice, with the exception of the levels of TNF-α secreted by the PAM, which were similar to those of the NPAM.

**Figure 8:**
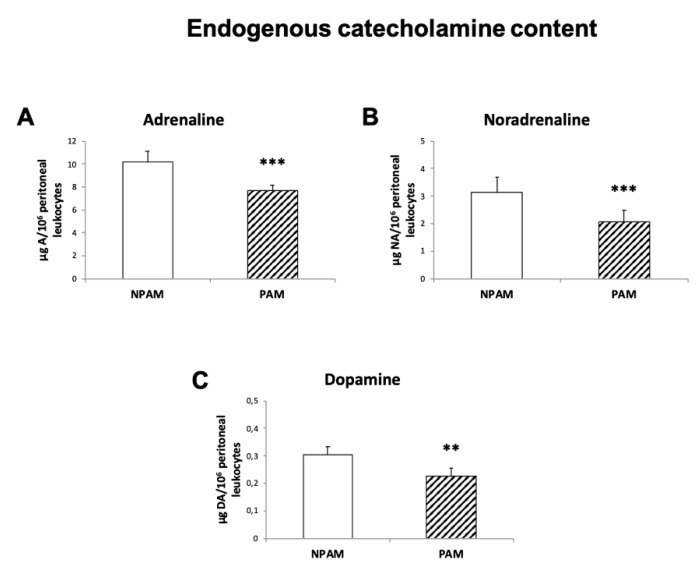
Endogenous catecholamine content (µg/10^6^ peritoneal leukocytes) analyzed in peritoneal leukocytes from PAM and NPAM. Adrenaline (**A**), Noradrenaline (**B**), and Dopamine (**C**) from adult PAM and NPAM. PAM: prematurely aging mice, NPAM: non-prematurely aging mice. Each column is the mean ± SD of the values of 10 experiments corresponding to 10 animals. ** *p* < 0.01, *** *p* < 0.001 with respect to NPAM.

Finally, several previous studies have described the importance of catecholaminergic homeostasis on immune function modulation, an effect mostly mediated by endogenous CA through paracrine/autocrine pathways [48,49,50,51]. In agreement with this, and with the objective to determine if the premature immunosenescence establishment observed in PAM at adult age could be related to lower CA amounts in their peritoneal leukocytes, we analyzed the endogenous content of three CA in this location from PAM and NPAM. As shown in Figure 8, PAM showed lower adrenaline, noradrenaline and dopamine levels in these cells in comparison to NPAM (*p* < 0.001; *p* < 0.001 and *p* < 0.01, respectively).

Therefore, all results obtained show a premature immunosenescence and oxidative and inflammatory stresses in the peritoneal leukocytes from adult PAM and TH-HZ mice, since, in the parameters analyzed, these cells present values similar to those in old animals. Furthermore, oxidative stress is also shown in other immunological organs, such as spleen and thymus. Moreover, PAM had lower CA amounts in their peritoneal leukocytes, similar to that which occurs in these cells from TH-HZ [35]. Thus, adult PAM and TH-HZ mice show an oxidative and inflammatory stress establishment, which is typical of aged animals, and could be the origin of the establishment of the immunosenescence observed in both premature aging models.

## 3. Discussion

We have proposed two models of premature aging in mice (PAM and TH-HZ animals) based on the immunosenescence shown at adult age, and confirmed by their lower lifespan in comparison with the corresponding controls (NPAM and WT mice, respectively). Although in several previous studies we have suggested the presence of an oxidative-inflammatory stress in peritoneal leukocytes from PAM and TH-HZ mice [10,33,35], the current work is the first in which this oxidative stress has been observed in three immune locations (peritoneal, spleen and thymus leukocytes). Moreover, since the values obtained in the parameters studied were similar to those in chronologically old animals, we have corroborated the premature oxi-inflamm-aging of these adult mice. In addition, in peritoneal leukocytes from mice of both models, the relation between their premature oxidative stress and immunosenescence has been established.

It has been proposed that immune cells could present an age-related loss in their capacity to regulate the oxidant and inflammatory compounds that they produce in order to carry out an adequate immune response, increasing the chronic oxidative stress of the organism [12,23,41,42,52]. For this reason, we analyzed several parameters of this stress in peritoneal leukocytes from adult PAM, NPAM, TH-HZ, and WT mice, and in those from chronologically old mice. Our results show that PAM and TH-HZ, at adult age, present higher oxidant compounds (XO activities, GSSG amounts, and GSSG/GSH ratios) and lower anti-oxidant defenses (CAT, GR, GPx activities, and GSH concentrations) in peritoneal leukocytes with respect to their corresponding controls (NPAM and WT animals). In previous studies, we have also observed lower values of CAT and GR activities in young PAM [53] and higher values of GSSG concentrations and GSSG/GSH ratios in adult PAM, with respect to NPAM [33,43,44]. Furthermore, in peritoneal leukocytes from adult TH-HZ mice, lower GR activity and GSH amounts and higher values of XO activity and GSSG concentrations together with higher GSSG/GSH ratios in comparison to their WT counterparts have also been found [35,45]. In the present work, we have corroborated that the oxidative stress of the peritoneal leukocytes from adult PAM and TH-HZ mice is similar to that in chronologically old mice. Furthermore, with the objective to determine whether the oxidative stress observed in peritoneal leukocytes from PAM and TH-HZ mice is also shown in other immunological organs, we analyzed the same anti-oxidant defenses and oxidant compounds in spleen and thymus leukocytes from these animals. Our results show that spleen and thymus leukocytes from PAM and TH-HZ presented higher oxidant compounds (XO activities, GSSG amounts, and GSSG/GSH ratio) together with lower anti-oxidant defenses (CAT and GR activities), with similar values to those of chronologically old mice. In another model of premature aging in mice, such as senescence-accelerated mouse prone (SAMP) 10, which mimics the age-related decline that occurs with chronological aging, an increased expression of reactive oxygen species in cells from thymus has been described [40]. This oxidative imbalance also has been described in spleen from SAMP8 mice [54]. This oxidative stress has also been reported in others tissues, such as brain, from these SAMP [55,56,57,58]. Thus, our results have proved that, at least for the parameters studied, the peritoneal immune cells, which can be obtained without sacrificing the animal, are very representative of other leukocytes and, possibly, of cells from non-immunological organs. A similar fact was observed in ovariectomized rats and mice, a model of accelerated aging, which mimics the lost estrogen of human menopause. In these animals, the oxidative stress detected in peritoneal leukocytes was also presented in immune cells from spleen and other organs [59]. Thus, peritoneal leukocytes seem to be useful for the study of the redox situation of the organism.

Another feature that occurs with age, associated to oxidative stress establishment, is the appearance of a low-grade inflammatory status due to the elevation of circulating amounts of pro-inflammatory cytokines such as TNF-α, IL-1β or IL-6, which are principally released by resting leukocytes [18,19,20,28,29,30,31,32,33,34,35,36,37,38,39,40,41,42,60]. This “inflamm-aging” forms part of the basis of immunosenescence [7,18,19,20,28,29,30,31,32,33,34,35,36,37,38,39,40,41,42,60,61], and has been related to a higher risk of mortality [62,63,64]. In this context it should be considered that when the immune cells carry out their functions, they produce inflammation, which is necessary for an adequate immune response against infections. However, when the inflammation is generated without the presence of pathogens, it is denominated “sterile inflammation.” This inflammation shares mechanisms with infectious inflammation and, although they are not identical processes, this sterile inflammation can produce damage in the organism [60,64]. In sterile inflammation, which increases with aging, cells of innate immunity, such as phagocytes and innate lymphoid cells (ILCs), are the most involved [65]. To assess this inflammation we have analyzed, in basal cultures of peritoneal leukocytes, the capacity of releasing several pro-inflammatory (IL-1β, IL-6 and TNF-α) cytokines. The results showed that these leukocytes from PAM and TH-HZ mice released higher pro-inflammatory cytokine amounts than those of their corresponding controls. Furthermore, the ratios of the pro-inflammatory/anti-inflammatory cytokines were higher, in general, in PAM and TH-HZ mice, than in the corresponding controls. Moreover, these ratios were also higher in cultures of peritoneal leukocytes from chronologically old mice. In agreement with our results, previous reports have described a similar pro-inflammatory pattern in plasma of other premature aging models, such as SAMP8 [66,67]. Furthermore, the balance between pro- and anti-inflammatory signals regulates inflammatory response, which can lead to either restoration of health, if this balance is recovered, or to the development and progression of disease, if this imbalance is maintained over the time [61,63,65]. In our case, the results seem to indicate an inflammatory stress in peritoneal leukocytes from adult PAM and TH-HZ, which is similar to that in old animals. Nevertheless, pro-inflammatory and anti-inflammatory cytokine levels in spleen and thymus leukocytes should be evaluated in future experiments with the objective of determining whether, in these immune locations, inflammatory stress is also established. Inflammation is always a parallel result of oxidation due to the feature that the overproduction of oxidants leads to signaling cascades that trigger the generation of pro-inflammatory cytokines, and inflammatory compounds, which in turn also induce oxidant production and the impairment of antioxidant systems, giving rise to oxidative stress [21,22,23]. The present results seem to support this idea, since we have observed an oxidative and possible inflammatory stress state in leukocytes of adult mice of both models of premature aging, similar to that in chronologically old animals. In addition, the resting lymphoproliferation could also be considered a useful parameter to detect sterile inflammation, given that it provides information on how much inner damage the immune system is trying to fight. In fact, there is an age-related increase in the basal proliferation of lymphocytes [18,19,20,37,39,40,41]. In this context, peritoneal leukocytes from PAM and TH-HZ mice showed higher resting lymphoproliferation with respect to that observed in their corresponding controls (NPAM and WT mice, respectively) and similar to that found in chronologically old animals.

Since oxidative-inflammatory stress has been proposed as the cause of immunosenescence [12,18,19,20,22,23,24], several relevant functions of peritoneal macrophages and lymphocytes, which show age-related changes [2,10,37,39,40,46,47] were analyzed in adult PAM and TH-HZ mice as well as in old animals. The results showed that, in peritoneal leukocytes of PAM and TH-HZ mice, the phagocytosis of macrophages and the lymphoproliferation in response to ConA presented lower values than in NPAM and WT mice, respectively, and, in general, similar to those in old mice. Although these lower values in peritoneal cells from adult PAM and TH-HZ mice with respect to their corresponding controls have been previously observed for some functions [10,33,35,43,44,45], the present experiment is the first in which the similarity between the values obtained in cells from PAM and TH-HZ with chronologically old mice, has been observed. A lower phagocytosis of peritoneal macrophages has been related to a higher morbidity and mortality [33]. It has been reported by many authors that T-lymphocyte proliferation in response to mitogens such as Con A, which mimics their stimulation by antigens [68,69], shows a sharp drop with aging [60,70], and that this decreased lymphoproliferative response is linked to an increased mortality. Moreover, this immune function belongs to the signature of the immune risk phenotype in humans [71,72]. Nevertheless, future experiments are needed in order to evaluate these immune functions in spleen and thymus leukocytes. With respect to the capacity of leukocytes from PAM and TH-HZ of releasing cytokines in the presence of a stimulus, such as mitogens, they showed, in general, lower capacity than cells of the corresponding controls, and similar to those in leukocytes of old animals. The secretion of IL-2, the cytokine triggered primarily by the activation of T cell, promoting expansion of this population of lymphocytes, declines with age [20,37,38,60]. Although in a previous study we observed lower IL-2 release of immune cells from PAM with respect to NPAM [43], the present work is the first in which this fact has been observed in cells from TH-HZ with respect to WT, and also the first comparing the values of IL-2 in both PAM and TH-HZ with those of old animals. We have observed similar amounts of this cytokines in PAM, TH-HZ and old mice. The other cytokines analysed in this context, with very relevant roles in the immune response to antigens, decrease their secretion in leukocytes from old mice [60]. The present work is the first in which these cytokines have been studied in cultures of leukocytes from PAM and TH-HZ mice, and in both cases the amounts released are, in general, lower than in the corresponding controls and similar to those in chronological old animals. For all these reasons, the presence of an impaired immune response in adult PAM and TH-HZ mice, is evident. Considering the relevance of an optimal immune function for successful aging, this premature immunosenescence could explain the shorter life span of PAM and TH-HZ mice in comparison to the corresponding NPAM and WT animals, which have been previously described [10,33,35]. Even so, future research related to typical senescence markers, such as telomere shortening/activity or p16 and p53 levels, are needed in order to clarify the relation between immunosenescence and the shorter lifespan observed in both premature aging models.

Interestingly, PAM and TH-HZ mice are characterized by their inappropriate responses to stress situations [34,35]. Stress, which has been defined as “the nonspecific response of the body to any demand”, forms part of life since all organisms are subjected continuously to “stressors” (all stimuli able to induce stress response). Thus, the adequate response to stressors is associated with health maintenance, but the failure of adaptation to life stress, causes disease [57,58]. In this context, central nervous system (CNS) and the sympathetic nervous system (SNS), as well as the neuroendocrine system, more specifically the sympathetic-adreno-medullar (SAM) and hypothalamic-pituitary-adrenal (HPA) axes, are involved in the maintenance of adequate stress response. In fact, neurotransmitters and hormones, such as noradrenaline and catecholamines as well as glucocorticoids, released by the SNS and the SAM and HPA axes, play an important role in maintaining physiological homeostasis, modulating immune functions, especially during a stress response [73]. In this context, our results show that PAM at adult age exhibit, in their peritoneal leukocytes, lower values of the three CA analyzed than NPAM. In a previous study we have described a similar pattern in the case of TH-HZ mice [45]. These results seem to indicate an altered stress response in adult mice, not only TH-HZ animals, but also in PAM. This fact also occurs in old individuals, and seems to be responsible for their decreasing stress adaptability, which contributes to their age-related health impairment [74,75,76,77]. Nevertheless, future experiments are needed in order to analyze other molecular mechanisms and senescence markers related to stress response. Furthermore, these animals with altered catecholaminergic homeostasis seem to present oxidative and inflammatory stresses, which associated with an immunosenescence, lead to a premature aging. In fact, several previous studies have described the importance of catecholaminergic homeostasis on immune function modulation, effects mostly mediated by endogenous CA through paracrine/autocrine pathways [48,49,50,51,52]. Thus, a disruption of this homeostasis is associated with immune function impairments [48,50,51,52]. The results obtained in the present work support this idea. Moreover, PAM, which are detected exactly by their inappropriate response to stress situations, such as the exploration of a new environment like a T-maze [34,35], are a good example of that proposed idea in a natural model. They show inappropriate response to stress, exhibiting lower peritoneal CA endogenous amounts together with a premature immunosenescence and early mortality [21,33,44]. Moreover, these animals, due to their natural premature aging, could allow more easily the extrapolation the results obtained with them to humans. Since in PAM some lifestyle strategies, such as the ingestion of diets with appropriate amounts of antioxidants [53,78,79,80], seem to control the premature immunosenescence and oxidative-inflammatory stress, these animals and the TH-HZ mice could be good models to study several potential interventions to improve the biological age of chronologically adult individuals Nevertheless, future experiments are needed in order to corroborate these hypotheses.

In conclusion, this work suggests that PAM and TH-HZ mice, at adult age, show an oxidative-inflammatory stress, which is associated to the appearance of premature immunosenescence, leading to a higher biological age and explaining their lower lifespan. Also, these animals exhibit lower endogenous peritoneal CA amounts, which could be responsible for immune function deteriorations, in part at least. Thus, an inadequate stress response at adult age seems to be associated with oxidative and inflammatory stresses and immunosenescence, increasing the biological age of individuals and, hence, lowering their lifespan.

## 4. Materials and Methods

### 4.1. Animals

Adult (36 ± 4 weeks of age) female CD1 mice (Janvier, Saint-Berthevin, France) classified as prematurely aging mice (PAM, *N* = 10) and non-prematurely aging mice (NPAM, *N* = 10) following a method previously described [19,20], were used. Ten adults female CD1 mice with the deletion of a single allele (Hemi-Zygotic; HZ) of Tyrosine Hydroxylase (TH) (TH-HZ) and 10 wild-type (WT) mice obtained from the laboratory of Dr. Flora de Pablo of the Biological Research Institute (CIB, CSIC, Madrid, Spain) were also employed. These TH-HZ mice come from C57BL6/J TH-HZ mice kindly provided by Dr. Palmiter [67] and crossed with wild-type CD1 mice for up to 10 generations. The TH-HZ mice were healthy and normal with no signs of any associated lesions. The growth rates of these animals were indistinguishable from those of WT mice, and normal reproduction was observed. Since inactivation of both *th* alleles of TH in mice results in mid-gestational lethality (about 90% of mutant embryos die between embryonic days 11.5 and 15.5) [81,82] we have used TH-HZ mice. In addition, 20 old (84 ± 4 weeks) female CD1 mice (Janvier) were used as age control animals (a group of 10 mice was analyzed in parallel to the PAM-NPAM and another of 10 mice to the TH-HZ and WT). All the mice were housed in groups (five individuals per group) in polyurethane boxes, at a constant temperature (22 ± 2 °C) and under a 12/12 h reversed light/dark cycle. The animals were fed with a standard A.04 diet (Harlam, Barcelona, Spain) pellets and tap water ad libitum. The project identification code is PROEX (373/15), which was approval by Complutense University of Madrid ethics committee (11/01/2016). 

### 4.2. Collection of Samples

#### 4.2.1. Peritoneal Leukocytes

Peritoneal suspensions from adult animals (10 PAM, 10 NPAM, 10 TH-HZ, and 10 WT) at 45 weeks of age, as well as from 20 old mice (84 ± 4 weeks) were obtained without sacrificing the animals. Briefly, each mouse was held by the cervical skin, the abdomen was cleansed with 70% ethanol and 3 mL of tempered sterile Hank’s solution were injected intraperitoneally. After massaging the abdomen, 90% of the injected volume was recovered. Then, the number of macrophages and lymphocytes from peritoneal suspensions were identified by their morphology and quantified in Neubauer chambers using optical microscopy (40×). Cellular viability, routinely measured before and after each experiment by the trypan-blue exclusion test, was higher than 95% in all experiments. The peritoneal suspensions were adjusted in the corresponding medium, to the specified number of macrophages, lymphocytes, or total leucocytes, depending on the parameter to be analyzed.

#### 4.2.2. Spleen and Thymus Leukocytes

Two weeks after the collection of peritoneal leukocytes, these 10 adult animals of each group as well as 10 chronologically old animals were sacrificed by cervical dislocation according to the guidelines of the European Community Council Directives 2010/63/EU. Spleen and thymus were aseptically removed, freed from fat and pressed gently through a mesh screen (Sigma-Aldrich, St. Louis, MO, USA) obtaining a cell suspension that was centrifuged to isolate the leukocytes. In the case of spleen, due to its high concentration of erythrocytes, centrifuging (1600 *g* for 10 min) in a gradient of Ficoll-Hypaque (Sigma-Aldrich) with a density of 1.070 g/mL was necessary to isolate leukocytes. Spleen and thymus leukocytes were counted in an optical microscope (40×) using a Neubauer chamber and adjusted to 10^6^ cells/mL in Hank´s solution or in a complete medium containing 1640 RPMI (PAA, Piscataway, NJ, USA) supplemented with gentamicine (10 mg/mL, PAA) and 10% heat-inactivated fetal calf serum (PAA). Cellular viability, routinely measured before and after each experiment by the trypan-blue exclusion test, was higher than 95% in all experiments. In the case of old mice, these animals not were sacrificed because they were used for other experimental procedures.

### 4.3. Oxidative Stress Parameters: Antioxidants and Oxidants, in Peritoneal, Spleen and Thymus Leukocytes

#### 4.3.1. Catalase Activity

Catalase (CAT) activity was determined in peritoneal, spleen and thymus leukocytes following a method previously described [83], with slight modifications introduced by our laboratory [66]. Aliquots of the three leukocyte samples adjusted to 1 × 10^6^ cells/mL in Hank´s solution were centrifuged at 1000× *g* for 10 min at 4 °C and the supernatants were removed. Thereafter, leukocyte pellets were re-suspended in 100 μL of 50 mM phosphate buffer 7.4 pH (Sigma-Aldrich), previously degassed. Then, cells were sonicated and centrifuged at 3200× *g* for 20 min at 4 °C. Aliquots of the supernatant were used to evaluate CAT. The enzymatic assay was followed using a spectrophotometer for 80 s at 240 nm through the decomposition of 14 mM hydrogen peroxide (H_2_O_2_; PANREAC, Barcelona, Spain), in phosphate buffer, into H_2_O and O_2_. The results were expressed as International Units (IU) of enzymatic activity per 10^6^ leukocytes.

#### 4.3.2. Glutathione Reductase Activity

Glutathione reductase (GR) activity was assessed in peritoneal, spleen and thymus leukocytes following a method previously described [84] with some modifications [85]. Aliquots of leukocytes adjusted to 1 × 10^6^ cells/mL in Hank´s solution were centrifuged at 1000 *g* for 10 min at 4 °C and the supernatants were removed. Thereafter, leukocyte pellets were resuspended in 200 μL of 50 mM phosphate buffer 7.4 pH (Sigma-Aldrich) plus 6.3 mM ethylene-diaminetetracetic acid (EDTA, Sigma-Aldrich), previously degassed. Then, cells were sonicated and centrifuged at 3200 *g* for 20 min at 4 °C, and aliquots of the supernatant were used to evaluate GR. This method is based on the oxidation of β-NADPH (β-nicotinamide adenine dinucleotide 2-phosphate reduced) (6 mM, Sigma-Aldrich) due to the reduction of GSSG (80 mM, Sigma-Aldrich) by GR. The reaction was followed by spectrophotometry at 340 nm for 240 s. The results were expressed as milliunits (mU) of enzymatic activity per 10^6^ leukocytes.

#### 4.3.3. Glutathione Content

Both oxidized (GSSG) and reduced (GSH) forms of glutathione in peritoneal, spleen and thymus leukocytes, were determined using a fluorimeter as previously described [85] with some modifications [45], adapted to 96-well plates. This procedure is based on the capacity of reaction that GSSG shows with o-phthalaldehyde (OPT, Sigma-Aldrich), a fluorescent reagent, at pH 12, and GSH at pH 8, resulting in the formation of a fluorescent compound. Aliquots of leukocytes adjusted to 1 × 10^6^ cells/mL in Hank´s solution were centrifuged at 1000 *g* for 10 min at 4 °C and the supernatants were removed. Thereafter, leukocyte pellets were re-suspended in 100 μL of 50 mM phosphate buffer pH 7.4 (Sigma-Aldrich) plus 0.1 mM EDTA (Sigma-Aldrich). Cells were sonicated and 400 μL of the specific buffer as well as 5 μL of 60% perchloric acid (HClO4; Sigma-Aldrich) were added. Immediately, samples were centrifuged at 9600 *g* for 10 min at 4 °C. Aliquots of the supernatant were used to evaluate both GSSG and GSH levels. Aliquots of 10 μL of supernatants of immune cells were dispensed into two 96-well black plates (Nunc, Roskilde, Denmark), one for each glutathione form. For GSSG measurement, 12 μL of N-ethylmaleimide 0.04 M (NEM, Sigma-Aldrich) were added to each well to prevent interference of GSH with measurement of GSSG, and the plate incubated at room temperature for 30 min in the dark. Then, 178 μL of NaOH 0.1N (PANREAC) and 20 μL of OPT (1 mg/mL in 99% methanol, PANREAC) were incorporated and the plate was incubated for 15 min under the same conditions. The fluorescence emitted by each well was measured at 350 nm excitation and 420 nm emission and the results were expressed as nmol/10^6^ cells. For the measurement of GSH content, 190 μL of phosphate buffer with EDTA and 20 μL of OPT were added to the 10 μL of cell supernatants dispensed in the wells. The plate was incubated for 15 min under the same conditions, and the fluorescence emitted by each well was measured at the same wavelength. The results were expressed as nmol/10^6^ cells. Also, GSSG/GSH ratios were determined.

#### 4.3.4. Xanthine Oxidase Activity

Xanthine oxidase (XO) activity was determined by fluorescence using a commercial kit (A-22182 Amplex Red Xanthine/Xanthine Oxidase Assay Kit, Molecular Probes, Paisley, UK). In the assay, XO catalyzes the oxidation of purine bases (xanthine) to uric acid and superoxide. The superoxide spontaneously degrades in the reaction mixture to H_2_O_2_, which in the presence of horseradish peroxidase (HRP) reacts stoichiometrically with Amplex Red reagent to generate the red-fluorescent oxidation product, resorufin. Aliquots of peritoneal, spleen and thymus leukocytes adjusted to 1 × 10^6^ cells/mL in Hank´s medium were centrifuged at 1000 *g* for 10 min at 4 °C and the supernatants were removed. Then, cell pellets were re-suspended in 100 μL of 50 mM phosphate buffer (7.4 pH) (Sigma-Aldrich) containing EDTA (1 mM), sonicated and centrifuged at 3200 *g* for 20 min at 4 °C. The assay was carried out in 50 μL of the supernatants incubated with 50 μL working solution of Amplex Red reagent (100 μM) containing HRP (0.4 U/mL) and xanthine (200 μM). XO (10 mU/mL) supplied in the kit was used as the standard, and XO activity was measured by comparing the fluorescence of samples with those of standards. After 30 min of incubation at 37 °C, measurement of fluorescence was performed in a microplate reader (Fluostar Optima, BMG Labtech, Biomedal, Madrid, Spain) using excitation at 530 nm and emission detection at 595 nm. The results were expressed as international milliunits (mU) of enzymatic activity per 10^6^ leukocytes.

### 4.4. Inflammatory Stress. Pro-inflammatory and Anti-Inflammatory Cytokines in Peritoneal Leukocyte Cultures

The levels of pro-inflammatory cytokines (TNF-α, IL-1β and IL-6) as well as the anti-inflammatory cytokine IL-10 were analyzed in the samples of 100 μL collected of peritoneal leukocyte cultures of 48 h in basal conditions, in presence of ConA (1 μg/mL) or lipopolysaccharide (LPS) (1 μg/mL), which were maintained at −80 °C until the measurement of the cytokines. The analysis of these cytokines was carried out using luminometry and a mouse cytokine/chemokine magnetic bead panel (Milliplex MAP kit, Millipore, Burlington, MA, USA). Briefly, the filter plate was pre-wet with assay buffer and vacuum filtered before adding 25 μL of standard (control) or samples to the appropriate wells. Then, 25 μL of premixed beads were added to each well and the plate incubated overnight at 4 °C with shaking. After two washes, 25 μL of detection antibody was added to each well and the plate incubated for 1 h at room temperature and then treated with 25 μL of streptavidin-phycoerythrin for 30 min at room temperature. The plate was washed twice with wash buffer and vacuum filtered and finally the beads were re-suspended in 150 μL of sheath fluid. The results were expressed in pg/mL. Concentrations as low as 2.3 pg/mL for TNF-α, 5.4 pg/mL for IL-1β, 1.1 pg/mL for IL-6 and 2.0 pg/mL for IL-10, were able to be detected. Additionally, TNF-α/IL-10, IL-1β/IL-10 and IL-6/IL-10 ratios were calculated.

### 4.5. Functions of Peritoneal Immune Cells

#### 4.5.1. Phagocytic Index

Phagocytic index (PI) was assayed according to the method previously described [10]. Aliquots of 200 μL of peritoneal suspension adjusted to 5 × 10^5^ macrophages/mL in Hank´s medium were placed on migratory inhibitory factor (MIF) plates (Kartell, Noviglio, Italy) and incubated for 40 min at 37 °C in a humidified atmosphere of 5% CO_2_. The adherent monolayer obtained was washed with pre-warmed phosphate-buffered saline (PBS) solution, and then 200 μL of Hank´s medium and 20 μL of latex beads suspension (1% in PBS medium; 1.1 μm mean particle size, Sigma-Aldrich) were added. After 30 min of incubation under the same conditions, plates were gently washed with PBS, fixed, and stained, and the number of latex particles ingested by 100 macrophages (phagocytic index) was determined by optical microscopy (100×).

#### 4.5.2. Lymphoproliferation

The proliferative capacity was evaluated as previously described [10]. Proliferation of lymphocytes both in basal conditions and in response to the mitogen Concanavalin A (ConA, Sigma-Aldrich) were assayed. Aliquots of 200 μL/well of peritoneal suspensions adjusted to 1 × 10^6^ lymphocytes/mL in the 1640 RPMI complete medium were incubated with 20 μL/well of complete medium (basal proliferation) or 20 μL/well of ConA (1 μg/mL) (proliferation in response to mitogen) were added. After 48 h of incubation at 37 °C in a sterile and humidified atmosphere of 5% CO_2_, 100 μL of culture supernatants were collected and to minimize oxidation, these aliquots were rapidly frozen and stored at −80 °C until the assays of cytokine levels were performed. Then, 7 μL ^3^H-thymidine (ICN, Costa Mesa, CA, USA) were added to each well (2,5 μCi per well), and the medium was renewed. The cells were harvested 24 h thereafter in a semi-automatic harvester (Skatron Instruments, Lier, Norway), and thymidine uptake was measured in a β counter (LKB, Uppsala, Sweden) for 1 min. The results were expressed as counts per minute (c.p.m.) in the case of basal proliferation. Regarding the results of the proliferative response to ConA, the percentage (%) of stimulation was calculated giving the value 100 to the cpm of the basal proliferation.

#### 4.5.3. IL-2 Release Measurement

In the samples of 100 μL of supernatants collected from the cultures of 48 h of peritoneal leukocytes in presence of ConA, the levels of IL-2 (and of the other cytokines described below) were measured by luminometry using a mouse cytokine/chemokine magnetic bead panel (Milliplex MAP kit, Millipore). The results were expressed in pg/mL. Concentrations as low as 1 pg/mL for IL-2, were detected.

### 4.6. Endogenous Catecholamine Content

Aliquots of 10^6^ peritoneal leukocytes/mL Hank’s solution, obtained from PAM and NPAM at adult age (45 weeks of age), were centrifuged at 1200 *g* for 10 min at 4 °C. Pelleted cells were re-suspended in HCl (0.01 N, Panreac) buffer in presence of EDTA (1 mM; Sigma-Aldrich) and sodium metabisulfite (4 mM; Sigma-Aldrich). Later, samples were sonicated and centrifuged at 3200 *g* for 20 min at 4 °C. CA levels were analyzed in the leukocyte supernatant with a commercially ELISA kit (3-CAT Research ELISA™, LDN Labor Diagnostika Nord, Nordhorn). Results were expressed as µg/10^6^ peritoneal leukocytes, and each sample was assayed in duplicate.

### 4.7. Statistical Analysis

The data were expressed as the mean ± S.E.M. of the values. Statistics were performed using SPSS version 21.0 (Chicago, IL, USA). The normality of the samples was tested by the Kolmogorov-Smirnov test. The data were statistically evaluated by Student´s *t* tests, and the minimum significance level established as *p* < 0.05.

## Abbreviations

PAM	Prematurely aging mice.
NPAM	Non-prematurely aging mice
TH-HZ	Tyrosine hydroxylase haploinsufficient mice
LPS	Lipopolissacharide
ConA	Concanavaline A
*th*	Tyrosine hydroxylase gene
TH	Tyrosine hydroxylase enzyme
A	Adrenaline
NA	Noradrenaline
DA	Dopamine
